# Exploring the association between smartphone-based place visitation data and neighborhood-level coronary heart disease in the United States

**DOI:** 10.1371/journal.pone.0329455

**Published:** 2025-08-14

**Authors:** Temitope Akinboyewa, Zhenlong Li, Huan Ning, M. Naser Lessani, Louisa M. Holmes, Shan Qiao

**Affiliations:** 1 Geoinformation and Big Data Research Laboratory, Department of Geography, The Pennsylvania State University, University Park, Pennsylvania, United States of America; 2 Departments of Geography and Demography, The Pennsylvania State University, University Park, Pennsylvania, United States of America; 3 Department of Health Promotion, Education, and Behavior, Arnold School of Public Health, University of South Carolina, Columbia, South Carolina, United States of America; Villanova University, UNITED STATES OF AMERICA

## Abstract

Coronary Heart Disease (CHD) is the leading cause of death in the United States, affecting over 20.5 million adults. Previous studies link health behaviors – such as dietary behavior, physical activity, smoking, and alcohol consumption – to CHD risk. These studies typically use surveys and interviews, which, despite their benefits, are resource-intensive and limited by small sample sizes. Using large-scale national level anonymized smartphone-based location data, our study examines whether health behaviors that are proxy measured by place visitation are associated with CHD prevalence across US census tracts. This study utilized data from multiple sources, including demographic and socioeconomic characteristics, health outcomes, and smartphone-based place visitation data. Health behavior measures were derived from aggregated smartphone location data at the census tract level, focusing on categories such as food retails, drinking places, and physical activity locations. Three sets of regression analyses were conducted: one using only demographic variables, the second including socioeconomic variables, and another incorporating the derived health behavior measures. Linear and spatial regression analyses were employed to assess the relationship between neighborhood-level CHD prevalence and these behaviors. Findings indicate a significant association between health behaviors that are proxy measured by place visitation data and the prevalence of CHD at the neighborhood level. The models incorporating these behaviors demonstrated improved fitness and highlighted specific behavioral factors such as increased visits to physical activity facilities and healthy food retail associated with lower CHD rates. Conversely, higher visits to less healthy food retail were associated with increased CHD rates. Smartphone-based visitation data offers a novel method to assess health behaviors at a large scale, providing valuable insights for targeting CHD interventions more effectively at the neighborhood level. This approach could enhance our understanding and management of CHD, informing public health strategies and interventions to mitigate this major health challenge.

## 1. Introduction

Heart disease is a serious public health challenge in the United States (US). The Centers for Disease Control and Prevention (CDC) report that heart disease is the leading cause of death in the US [1]. Heart disease was responsible for one out of every five deaths in the US in 2021 [2]. Coronary heart disease (CHD) is a prevalent type of heart disease, which happens when the arteries cannot provide adequate oxygen-rich blood to the heart. In 2021, CHD was responsible for causing the highest number of deaths (40.3%) attributable to Cardiovascular Disease (CVD) in the US [3]. As of 2023, approximately 20.5 million American adults had been diagnosed with CHD [4].

Research indicates that demographic factors and socioeconomic status (SES) are closely linked to CVD risk. Age significantly influences CVD risk due to physiological changes and accumulated risk factors, with higher prevalence in older populations [5,6]. Also, lower SES, often measured by income, education, and occupation, is consistently associated with higher CVD incidence and prevalence. This relationship is attributed to limited healthcare access, delaying diagnosis and treatment [7,8], less access to healthy food and safe environments for physical activity, contributing to obesity and hypertension, both of which are risk factors for CVD [9,10], and higher stress levels associated with financial instability and job insecurity, exacerbating cardiovascular risk [11].

Past studies have demonstrated a correlation between individual behaviors and the likelihood of developing CHD. For instance, the consumption of healthy foods such as fruits and vegetables has been found to have a substantial negative relationship with the risk of heart disease [12–14]. These food groups are rich in vitamins, minerals, and antioxidants, which play crucial roles in heart health by reducing blood pressure and mitigating inflammation [15]. In contrast, a diet high in fast foods, processed meats, and sugary snacks is linked to negative impacts on cardiovascular health, leading to obesity and high blood pressure, which can increase the risk of CHD [16–18]. On the other hand, frequent visits to healthy food retails, such as fruit and vegetable stores and grocery stores, are inversely related to the prevalence of CHD, while frequent visits to unhealthy food retails, such as convenience stores and limited-service restaurants, are positively related to the prevalence of CHD.

Physical activity is another critical factor in reducing the risk of CHD [19]. Studies have demonstrated that regular exercise, such as park visitation and engagement in recreational and sports activities, can significantly improve cardiovascular health [20,21]. These activities help maintain a healthy weight, reduce cholesterol levels, and strengthen the heart muscle. Therefore, it can be theorized that visits to places promoting physical activities, like fitness centers and recreational sports facilities, are inversely related to the prevalence of CHD. Additionally, smoking and excessive alcohol consumption are associated with increased CHD risk. Smoking has a direct negative impact on heart health [22]. While moderate alcohol intake might have some protective effects [23], excessive drinking can lead to heart failure, high blood pressure, and obesity [24]. Thus, it can be inferred that there is a positive relationship between the prevalence of CHD and the visit rate to establishments associated with alcohol consumption and smoking, such as tobacco stores and drinking venues.

Despite the strong association between CHD and health behaviors, measuring these behaviors can be challenging. Researchers often rely on survey data and interviews to gather information. Surveys and interviews are valuable as they provide specific insights into the characteristics of individuals that may influence health outcomes. They provide detailed data that can be used to create personalized health strategies and guidance. However, this approach is limited in several ways. First, it is financially costly and demands substantial labor resources, making it impractical on a large scale. Moreover, the time required to conduct longitudinal studies, such as cohort and prospective studies, can range from months to years. As a result, it takes time to conduct data inputs, cleaning, deidentifying, and managing using the traditional approach. This can hinder the timely implementation of health interventions, and policies that are aimed at addressing emerging health issues. Also, due to coordination and data ownership challenges, conducting a large-scale cross-region survey to cover large populations in diverse geolocations is hard. As a result, research studies that rely on conventional methods are typically limited to a specific geolocation [25]. In addition, traditional surveys and interviews often lack detailed spatial data, such as specific locations where individuals live, work, or engage in health-related behaviors, limiting the ability to analyze how geographical factors influence health behaviors and outcomes. When geographic data is included, it is often aggregated at a high level (e.g., state or city), which may obscure significant variations and contextual factors at more granular levels (e.g., neighborhoods or blocks). Furthermore, traditional methods may overlook environmental determinants of health, such as access to green spaces and healthy food options, which can significantly impact health behaviors and outcomes.

To overcome these limitations, an alternative approach that leverages neighborhood-level data aggregation emerges as a promising solution. Neighborhood-level data aggregation allows for the analysis of large populations across diverse geolocations without extensive on-the-ground survey efforts, which enables more comprehensive and inclusive studies that cover a wide range of communities. Also, by aggregating data at smaller geographic units, researchers can identify variations and patterns that are not apparent at higher levels. This granular analysis helps understand the specific contextual factors that influence health behaviors in different areas and identifies various factors contributing to health outcomes and their spatial connections [26]. Such information can help policymakers design targeted interventions and policies tailored to the unique needs of different neighborhoods.

Human mobility data derived from mobile devices, such as smartphones, have the potential to provide valuable insights into health behaviors by revealing place visitation patterns [27]. These patterns show where and how often people engage in various activities, which can help better understand the prevalence of health outcomes. Since the COVID-19 pandemic, there has been a rise in the use of anonymous location data from smartphones. Companies like SafeGraph collect data from various mobile devices and aggregate them into geographical areas, such as block groups. This aggregated data provides information about the mobility pattern of people in the geographic area, i.e., how people move around and interact with different places, referred to as points-of-interests (POIs). These POIs include restaurants, parks, recreational centers, health services, convenience stores, and supermarkets. Therefore, it is possible to use proxy measures, such as place visitation data, to assess health behaviors related to CHD.

While prior research has utilized place visitation data to evaluate how people’s health behaviors are related to different health outcomes [26,28–30], little attention has been given to understanding how aggregate behavioral patterns at the neighborhood level may relate to neighborhood-level CHD prevalence. Understanding such associations at the neighborhood level is important because CHD is strongly influenced by lifestyle factors that are reflected in patterns of place visitation. Traditional methods for capturing these behaviors often fail to provide scalable insights at the neighborhood level.

Therefore, in this study, we examine how health behaviors (i.e., place visitation rate) derived from mobile phone-based place visitation data can explain the prevalence of CHD at the neighborhood (census tract) level. To achieve this, we addressed the following research questions: 1) what is the feasibility of utilizing mobile phone-based place visitation data to proxy measure relevant health behaviors? and 2) how are proxy measures of health behaviors associated with the CHD prevalence controlling other population levels variables? Our research contributions are two-fold: first, this is the first nationwide study to explore how health behaviors contribute to the prevalence of CHD at the neighborhood level using mobile phone-based place visitation data; second, the findings offer insight into the importance of health behavior measures, such as rate of place visitation in different neighborhood-level CHD cases, which could guide interventions and predict CHD. The remainder of this paper is structured in the following way. In Section 2, we present the data and methods used to access CHD-related health behaviors, along with the experiments conducted to investigate how these behaviors influence the prevalence of CHD throughout the US. Section 3 provides a detailed analysis of the results obtained from the experiments, while Section 4 examines the implications of these findings. Finally, in Section 5, we offer a summary of the study.

## 2. Method

### 2.1 Data sources

This study employed three data sources: American Community Survey (ACS) 5-year Census data, Centers for Disease Control (CDC) Population Level Analysis and Community Estimates (PLACES) data, and SafeGraph Place visitation data. A total of 64,448 census tracts were included in this study, with tracts having any missing data in either the SafeGraph or PLACES datasets excluded to ensure data completeness.

The ACS covers a wide range of information about the US population’s demographic, social, and economic characteristics. The ACS 5-year estimates are reliable estimates that represent statistically representative data collected over a period of time. This is particularly useful for areas with smaller populations and subgroups [31]. In this study, the 2019 ACS 5-year estimates census data was used.

The CDC PLACES dataset provides health-related information for small regions throughout the US. It provides health estimates and analysis at population levels for regions like ZIP Code Tabulation Areas (ZCTA), census tracts, places, and counties, using a model-based approach [32]. This study utilized the census tract-level dataset for the 2021 release of the PLACES data. This release includes model-based estimates generated from the Behavioral Risk Factor Surveillance System (BRFSS) data containing 22 health measures, Census Bureau 2010 population data, and the ACS 2015–2019. The CHD prevalence was obtained from the CDC PLACES dataset. The CHD prevalence was quantified as the proportion of survey respondents aged 18 years or older who report having been diagnosed with CHD by a physician, nurse, or other healthcare professional [32].

SafeGraph is a company that provides combined and anonymized location data on POIs and visitation patterns from mobile devices, such as cell phones. SafeGraph provides information on visitation to millions of POIs across the US. These POIs include restaurants, recreational centers, markets, drinking places serving alcoholic beverages, and various stores, all classified according to the standards of the North American Industry Classification System (NAICS). The SafeGraph smart mobile device locations were gathered from over 45 million mobile phones [33]. SafeGraph data represents approximately 7.5% of the population on average, with notable variations in sampling rates across different geographic and temporal dimensions [27]. The data tends to be more representative of urban areas compared to rural ones, with a strong correlation between the number of sampled devices and the census population at the county level, particularly in urban counties (r > 0.97) and to a lesser extent in rural counties (r > 0.91) [27]. Also, the data exhibits minor biases in terms of gender, age, and moderate-income groups, with biases typically ranging from −0.05 to +0.05. However, there is a notable underrepresentation of minority groups such as Hispanic populations, low-income households, and individuals with low education levels, which varies across different spatial and temporal dimensions [27]. The visitation data used was weekly visitation pattern information, consisting of visits to various places in 2019.

To mitigate the bias, we applied a normalization approach that adjusts visit counts based on the number of mobile devices residing in each census tract. This method corrects for variations in SafeGraph sampling rates and ensures comparability across census tracts. Also, it is worth noting that some census tracts had missing data, the proportion was relatively small, and the normalization technique substantially minimized potential biases.

### 2.2 Overview of the study framework

**Fig 1:**
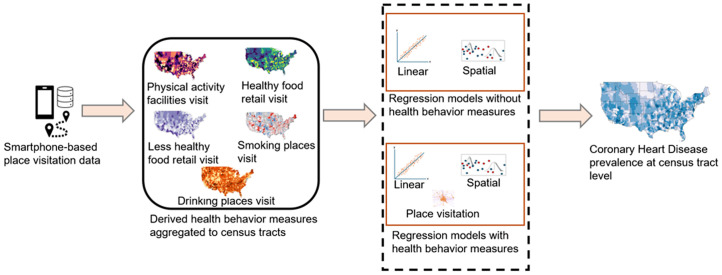
Overview of the study framework. All maps were created using ArcGIS Pro v3.3 by the authors.

This study aims to examine the extent to which health behaviors estimated from smartphone location data can explain the prevalence of CHD at the tract level. Fig 1 shows the overview of the study. First, health behavior measures (e.g., POI visitation) were estimated from smartphone-based place visitation data and aggregated at the census tract level. Specifically, we focused on five categories of place visitation: physical activity facilities, less healthy food retail, healthy food retail, drinking places, and smoking places. These categories were selected based on existing literature. More details about the categories of place visits are provided in Section 2.3. Subsequently, three sets of regression analyses were conducted to assess the explanatory power of the derived health behaviors. In the first regression model, demographic variables were used as independent variables, in the second model, socioeconomic variables were added, in the third model, the derived health behaviors were added to the demographic and socioeconomic variables. Subsequently, the models (with and without health behaviors) were compared in terms of their capacity to explain the variance of CHD prevalence.

### 2.3 Deriving place visitation rate as a proxy measure of health behaviors

Based on the existing literature, this study considered health behaviors related to CHD, including dietary habits [14,34,35], physical activity [19–21,36], and smoking and alcohol consumption [23,37]. The place visitation data was used as a proxy measure of these health behaviors. The first step involves the identification of the POIs associated with each category of health behaviors (Table 1). This was achieved in three ways. First, we followed literature that has used place visitation data as a proxy of human behavior to the same categories. Secondly, for health behaviors in which place visitation data has not been widely used as a proxy, we identified POI categories that are theoretically linked to relevant behaviors based on prior literature. For instance, golf courses and country clubs were categorized under physical activity, as they provide opportunities for physical engagement and have been associated with reduced cardiovascular risk [38]. Thirdly, the NAICS descriptions of POIs were reviewed to understand the description of the selected POIs to ensure accurate classification and avoid misinterpretation of place categories. It is worth noting that the selected POI categories capture only specific aspects of human behaviors and do not encompass all possible locations where people may engage in these behaviors. For example, while visits to fitness centers provide insights into key lifestyle choices, they do not fully account for other venues where people may exercise (e.g., public transit stations). Specifically, for dietary behaviors, we identified four types of POIs: fruit and vegetable markets [15], limited-service restaurants [39], convenience stores [40], supermarkets and grocery stores [28,41]. For physical activity, the identified POIs were fitness and recreational sports centers [42]. For tobacco and alcohol places, we identified beer, wine, and liquor stores [43], tobacco stores [44,45], and drinking places (serving alcoholic beverages) [46].

**Table 1:** Selected POIs categories related to CHD.

Place categories	POIs	References
Physical activity facilities	Fitness and recreational sports centers; Nature parks and other similar institutions; Golf courses and country clubs	[42,38]
Less healthy food retail	Convenience stores, Limited-service restaurants	[39,40]
Healthy food retail	Full-service restaurants, Fruit and vegetable markets, Supermarkets and grocery stores	[15,28,41,47]
Drinking places	Drinking places (alcoholic beverages); Beer, wine, and liquor stores, wineries, breweries	[46,43]
Smoking places	Tobacco store	[44,45]

After the identification of the relevant POIs, visits to the selected POIs were derived from SafeGraph weekly visitation dataset at the Census Block Group (CBG) level for 2019. This dataset includes the number of visits to each POI within each CBG on a weekly basis. The data was subsequently aggregated to the census tract level, where all visits to POIs within the same place category were summed together. The place visitation rate for each census tract was then calculated as summarized in Equation 1.


$${\text{Place\textbackslash{} visitation\textbackslash{} \textbackslash{} rate\textbackslash{} for\textbackslash{} census\textbackslash{} tract}}_{\text{t}}=\frac{\sum_{\text{i}=1}^{\text{n}}{\text{V}}_{\text{pt}}}{{\text{N}}_{\text{t}}}$$ (1)

Where ${\text{V}}_{\text{pt}}$ is the total number of visits to a type of POI p (e.g., convenience store); ${\text{N}}_{\text{t}}$ is the total population of tract tnumber of mobile devices in a census tract t and n is the total number of census tracts in the study area (n=64,448). It is important to note that census tract residents may visit POI types within or outside their neighborhood. Therefore, our methodology assigns visits to the home census tract of the visitor rather than the census tract where the POI is located. This means that the visitation rate for a given census tract reflects the aggregated visits made by its residents to relevant POIs, regardless of the POI location. This approach ensures that the visitation rate represents the behavioral tendencies of residents rather than the density of POIs in their home tract.

### 2.4 Demographic and socioeconomic variables

Past research has indicated that demographic characteristics and social and economic status are associated with CVD including heart disease [7,48,49]. In this study, we include measures about race and ethnicity (proportion of Black individuals, Hispanic, and Asian), age (percentage of those aged 65 and above), income (median household income), and social disadvantage level (ratio of individual living under the poverty level, the ratio of the uninsured population, and the ratio of the population who are unemployed. All the demographic and socioeconomic variables used (Table 2) were obtained from the 2019 ACS 5-year estimate data of the US census.

**Table 2:** Demographic and Socioeconomic variables used.

Variables	Descriptions
Demographic	
% 60 and above	Percentage of population equal or over age 60
% black	Percentage of the population who are Black
% hispanic	Percentage of the population who are Hispanic
% asian	Percentage of the population who are Asian
Socio-economic status	
% poverty	Percentage of the population below the poverty line
% uninsured	Percentage Civilian noninstitutionalized population with no health insurance covered
% unemployed	Percentage of the population who are not employed
Median household income	Median household Income

### 2.5 Statistical analysis

We conducted a correlation analysis to explore the general relationship between the prevalence of CHD, demographic, socioeconomic factors, and health behavior variables (S1 Fig). Then, we examined whether multicollinearity exists among the independent variables. To achieve this, the independent variables’ variance inflation factor (VIF) was computed, and the VIF for all the independent variables are all smaller than 5, indicating low multicollinearity among the variables (S1 Table). To observe the explanatory power of the independent variables, we performed a linear regression analysis with the CHD prevalence as the dependent variable and demographic variables, social economic variables, and health behaviors as the independent variables. The adjusted $R^{2}$, Akaike’s information criterion (AIC), and Bayesian Information Criterion (BIC) measures are used to compare model goodness-of-fit. The adjusted $R^{2}$ was used as a key evaluation metric because it accounts for the number of predictors in the model, providing a more reliable measure of explanatory power than the standard $R^{2}$ [50]. AIC takes into account both the model’s fit and its complexity, with lower AIC values indicating a superior model. Similarly, BIC places greater emphasis on model complexity compared to AIC. Therefore, the lowest BIC value typically identifies the simplest model that still effectively fits the data. Python’s statsmodels library for OLS was utilized for the linear regression. We further conducted an interaction analysis. This analysis specifically examined interactions involving age and income, as they demonstrated the strongest positive and negative relationships, respectively, among all demographic and socioeconomic variables with CHD prevalence (S1 Fig).

To understand the spatial distribution of CHD and the independent variables with the aim of detecting patterns of clustering or dispersion, we conducted spatial autocorrelation analysis. This was achieved by using the Global Moran’s I statistic [51], a statistic that quantifies the relationship between variables and their spatial positions. The reasoning behind checking for spatial autocorrelation is the First Law of Geography [52]. Spatial data, like census tract-level variables, frequently show spatial dependence, which contradicts the assumption of independent measurements [53]. The Moran’s I values indicated a significant spatial autocorrelation in the data, which suggests that spatial regression analysis could be employed. We then considered the spatial lag model and spatial error model to account for the impact of spatial correlation. Prior to selecting the spatial regression model, Lagrange multiplier test was performed to evaluate the type of spatial dependence in the data and robust LM tests were developed to help in selecting the best spatial regression model. Following the test, the spatial error model was considered the most suitable model compared to spatial lag model. Therefore, spatial error model was fitted using a maximum likelihood (ML) estimation approach, with spatial weights generated based on queen contiguity criteria. The SEM was implemented using Python’s PySAL library.

## 3. Results

### 3.1 Spatial distribution of the neighborhood-level CHD prevalence and health behavior measures

**Fig 2:**
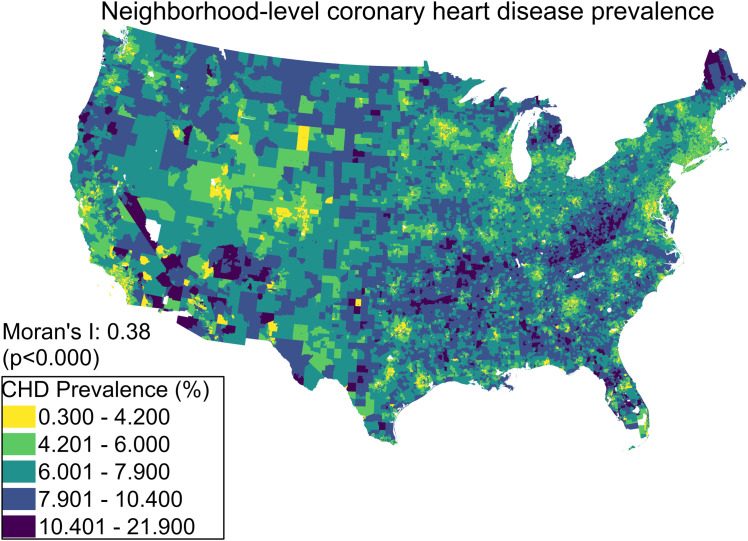
Neighborhood-level CHD prevalence in the US. Map was produced using ArcGIS Pro v3.3 by the authors.

Fig 2 visualizes the 2019 CHD prevalence at the census tract level in the US. The CHD prevalence tends to be clustered across the US and varies across different regions in the US. Higher prevalence is observed mostly in the Southeastern US (West Virginia, Kentucky, Arkansas and Oklahoma), Northeastern states (Maine), some areas in the Western US (California, Oregon, and Arizona), and Northern US (Montana). Given this observation, the spatial autocorrelation was examined using the global Moran’s I index. We found that the CHD prevalence has a statistically significant and positive spatial autocorrelation, with a Moran’s I index of 0.38 (p < 0.05).

**Fig 3:**
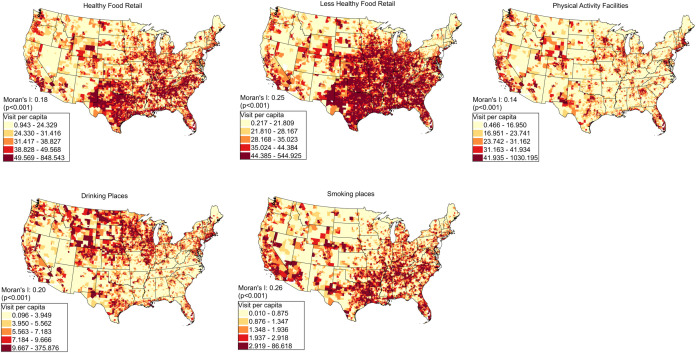
Spatial distribution of the five health behavior measures (place visitation rate) at census tracts level in the US. Visitation rate is the total number of visits to a type of POI per total number of mobile devices in a census tract. All maps were produced by using ArcGIS Pro v3.3 by the authors.

Fig 3 presents maps of place visitation rates of the census tracts in the contiguous US to various POIs. Overall, the maps reveal distinct regional place visitation patterns and the Moran’s I indices for all POI visits reveal positive and statistically significant values (p < 0.05), suggesting the presence of significant spatial autocorrelation in the visitation rates of mobile devices in each tract to the POIs. For the Healthy Food Retail category, the visitation rates are highest across the regions along the East Coast, the West Coast, and Southern regions, with a moderate level of spatial clustering (Moran’s I: 0.18, p < 0.000). The visitation rate of Less Healthy Retail category is highest in the Southeastern and South-central regions, with a moderate to strong pattern of clustering (Moran’s I: 0.25, p < 0.000). For Physical Activity Facilities, the visitation rates are high across diverse regions, particularly in the Western, Northeastern, and some Southeastern areas of the US. The spatial clustering reveals a relatively low spatial autocorrelation (Moran’s I: 0.14, p < 0.000), suggesting that the overall distribution is somewhat scattered across the US. Drinking places have the highest visitation in the Central and Northern regions, with moderate clustering (Moran’s I: 0.20, p < 0.000). Smoking stores show high visitation in the Southeastern, Central, and some Western regions, with moderate clustering (Moran’s I: 0.26, p < 0.000).

### 3.2 Statistical analysis results

#### 3.2.1 Correlation analysis results.

The Pearson correlation analysis (S1 Fig) indicates that all the demographic and socioeconomic status variables exhibit correlations with the outcome variable. Particularly, the percentage of population of age 60 years and above shows the highest positive correlation, while median household income shows the strongest negative correlation with the prevalence of coronary heart disease. Additionally, visitation rates to different categories of places demonstrate moderate correlations with CHD and among themselves, suggesting some level of interdependence. Following that, the variance inflation factor (VIF) analysis result (S1 Table) confirms the absence of multicollinearity among variables, indicating that each variable has a unique contribution to explaining CHD prevalence.

#### 3.2.2 Linear regression results.

Ordinary least squares (OLS) models were utilized for linear regression. Three models were fitted on the dataset. The first model consists of only demographic variables as independent variables. The second model consists of socioeconomic status variables in addition to the demographic variables, while the third includes place visitation rates. As seen in Table 3, incorporating place visitation rates into the model led to a notable improvement. The adjusted $R^{2}$ increased from 0.710 to 0.730, indicating that the inclusion of place visitation variables enhanced the model’s ability to explain variance in the dependent variable. This improvement is further supported by decreases in the Akaike Information Criterion (AIC) and Bayesian Information Criterion (BIC), which dropped from 210,201–206,161 and from 210,284–206,289 respectively. These reductions suggest that the added complexity of Model 3 is justified by its improved performance. Additionally, the F-test was performed to compare the model improvement observed. It was found that the improvement in the model fit is statistically significant (p < 0.05).

**Table 3:** Evaluation metrics of the linear regression models.

Model	OLS Model Evaluation			F-Test for Model Comparison					
	Adj. $R^{2}$	AIC	BIC	df_resid	ssr	df_diff	ss_diff	F	Pr(>F)
1	0.447	255,139	255,185	69442	160198	0.0	–	–	–
2	0.710	210,201	210,284	69438	83865	4.0	76333	16748	0.000
3	**0.730**	**206,161**	**206,289**	69433	79113	5.0	4752	834	**0.000**

The regression coefficients derived from the OLS model were further analyzed. A summary of the information is presented in S2 Table.

Although the OLS regression models provided insights into the associations between CHD prevalence and place visitation variables, significant spatial autocorrelation exists in the variables. Therefore, spatial regression models (spatial lag model and spatial error model) were evaluated to explicitly account for this spatial dependence. The evaluation metrics for the spatial regression models are presented in S3 Table. Robust LM tests diagnostics suggested the spatial error model as the superior model due to its higher robust LM statistic compared to the Spatial Lag Model.

The spatial error model results are presented in Table 4. From Model 1 (Table 4), the percentage of the population aged 60 and above is significantly and positively associated with higher CHD prevalence (coeff. = 1.220, p < 0.001). For racial and ethnic composition, a higher proportion of Black and Hispanic populations in a neighborhood is positively associated with CHD (coeff. = 0.024, p < 0.001 and coeff. = 0.010, p < 0.001, respectively), while the Asian populations show a significant negative association (coeff. = −0.507, p < 0.001). Among socioeconomic variables, from Model 2 (Table 4), higher level of poverty, unemployment and uninsured rate are significantly associated with increased CHD prevalence (coeff. = 0.296; 0.097; 0.579, p < 0.001 respectively), while median household income is strongly and negatively associated (coeff. = −0.482, p < 0.001). The results in Model 3 (Table 4) indicate that significant relationships exist between place visitation and CHD prevalence. Higher visitation to less healthy food retails is associated with increased CHD prevalence (coeff. = 0.114, p < 0.001), while higher visitation to physical activities places and healthy food retail is associated with lower CHD prevalence (coeff. = −0.009; −0.051, p < 0.001 respectively). Additionally, visitation to drinking shows a significant negative association with neighborhood-level CHD (coeff. = −0.209, p < 0.001) and smoking places shows non-significant negative association. The spatial error term is an additional indicator for the spatial error model. The coefficient of the error term measures the extent to which the model’s residuals (errors) are correlated across spatial units. The significant positive effect of the spatial error term in all three models confirms the presence of spatial correlation in the dataset.

**Table 4:** Spatial error model regression results showing associations between demographic variables, socioeconomic variables, place visitation behavior, interaction terms, and coronary heart disease (CHD) prevalence at census tract level.

	Model 1		Model 2		Model 3		Interaction analysis	
	Coeff.	Std. Error	Coeff.	Std. Error	Coeff.	Std. Error	Coeff.	Std. Error
**Demographic variables**								
%60 and above	0.1220***	0.001	1.2500***	0.005	1.2520***	0.005	**1.2852** ^***^	0.005
%black	0.0240***	0.000	0.0260***	0.006	0.0230***	0.006	0.0028	0.006
%hispanic	0.0100***	0.000	−0.1860***	0.006	−0.1820***	0.006	**−0.1687** ^***^	0.006
%asian	−0.0500***	0.001	−0.2560***	0.005	−0.2380***	0.005	**−0.2125** ^***^	0.005
**Socioeconomic variables**								
%uninsured			0.5790***	0.006	0.5990***	0.006	**0.5537** ^***^	0.006
%poverty			0.2960***	0.006	0.2710***	0.006	**0.2217** ^***^	0.005
%unemployed			0.0970***	0.005	0.0940***	0.005	**0.0825** ^***^	0.004
Median household income			−0.4820***	0.006	−0.4190***	0.007	**−0.5571** ^***^	0.007
**Health behavior measures**								
Physical Activities places					−0.0090***	0.005	**−0.0393** ^***^	0.006
Less healthy food retails					0.1140***	0.009	**0.1462** ^***^	0.009
Healthy Food retailer					−0.0510***	0.011	**−0.0993** ^***^	0.011
Drinking places					−0.2090***	0.007	**−0.2853** ^***^	0.007
Smoking places					−0.0092***	0.005	**0.0206** ^***^	0.005
**Interaction term**								
Physical Activities places * %60 and above							**−0.0695** ^***^	0.006
Physical Activities places * med_household_income							**0.0637** ^***^	0.006
Less healthy food * %60 and above							**0.2624** ^***^	0.009
Less healthy food retails * med_household_income							**−0.0883** ^***^	0.008
Healthy Food retailer * %60 and above							**−0.2627** ^***^	0.012
Healthy Food retailer * med_household_income							**0.2547** ^***^	0.011
Drinking places * %60 and above							**0.1547** ^***^	0.006
Drinking places * med_household_income							**0.0872** ^***^	0.008
Smoking places * %60 and above							**0.0314** ^***^	0.005
Smoking places * med_household_income							**−0.0549** ^***^	0.005
Spatial error term (λ)	0.3523***	0.005	0.3511***	0.005	0.3450***	0.005	**0.3453** ^***^	0.005
**Model Fit Criteria**								
Pseudo $R^{2}$	0.445		0.709		0.725		0.754	
Akaike Information Criterion (AIC)	228412.365		192071.760		189750.783		184412.371	
Bayesian Information Criterion (BIC)	228458.107		192154.095		189878.860		184631.931	

Note: * p < 0.05, ** p < 0.01, *** p < 0.001.

#### 3.2.3 Interaction analysis.

The negative association observed from the drinking places and smoking places variable was unexpected. Therefore, interaction analysis was carried out to account for some potential factors, particularly age and income, since these factors show very high correlation with CHD prevalence (S1 Fig). As shown in Table 3, the negative association between visitation to physical activity places and CHD prevalence is more pronounced in census tracts with a higher percentage of older adults (Physical Activity Places * %60 and above: coef. = −0.0695, p < 0.001), while this association is less negative in higher-income tracts (Physical Activity Places * Median Household Income: coef. = 0.064, p < 0.001). Conversely, the positive association between less healthy food retailer visitation and CHD prevalence is amplified in tracts with a higher percentage of older adults (coef. = 0.262, p < 0.001) but is mitigated in higher-income areas (coef. = −0.088, p < 0.001). A similar pattern is observed for healthy food retail, where their negative association with CHD prevalence is stronger in tracts with more older adults (coef. = −0.263, p < 0.001) and weaker in higher-income areas (coef. = 0.255, p < 0.001). For drinking and smoking behaviors, positive association between visitation to drinking places and CHD prevalence is more pronounced in older populations (coef. = 0.155, p < 0.001) and higher-income areas (coef. = 0.087, p < 0.001). The interaction effects for smoking places show that the association with CHD prevalence is amplified in tracts with older populations (coef. = 0.031, p < 0.001) but mitigated in wealthier areas (coef. = −0.055, p < 0.001).

## 4. Discussion

This study examines the association between health behavior measures derived from place visitation data in explaining CHD at the census tract level across the US. To achieve this, place visitation data was utilized to assess five neighborhood-level health behaviors and investigate their impact on enhancing the model that explains the prevalence of CHD. The study suggests that incorporating mobile phone-based place visitation data can improve the explanatory power of models that explain the prevalence of neighborhood-level CHD. Although, from the OLS regression analysis the improvement is minimal, as indicated by the Adjusted $R^{2}$ values, the substantial decreases in the AIC and BIC of the models, when the place visitation rates are included, emphasize the value of mobile phone-based data in modeling CHD prevalence. 

The geospatial patterns of place visitation rates in this study reveal significant regional variations and clustering tendencies. For example, the localized high visitation rates to fitness and recreational centers, as well as drinking places in the Western region (such as California) and Mid-west (such as Illinois), are consistent with the previous studies [30,46]. The robust economy of these regions and higher income levels are likely to contribute to the increased visitation rate of these POIs. Similarly, the rates of visitation to places that encourage unhealthy diets (such as convenience stores) are highest in the central region (such as Illinois and Iowa) and some eastern regions (such as New York, Washington DC, and Maryland). Zhou et al. (2022) [26] has also reported a high fast-food restaurant visitation rate in some parts of New York.

Regression analysis results reveal that census tracts’ demographic and socioeconomic characteristics are the primary factors contributing to the prevalence of CHD at the US census tract level. For instance, the percentage of the population aged 60 and older living in a census tract was related to a higher prevalence of CHD, consistent with previous research indicating that seniors may have a higher CHD prevalence [54,55]. Also, variables related to poverty status, such as the proportion of people living under the poverty line, unemployed, and uninsured, show a significant positive coefficient for neighborhood-level CHD. This aligns with previous studies that explore the association between poverty and heart disease [56]. Conversely, the median household income was found to be negatively associated with CHD prevalence. This suggests that higher income levels within the neighborhood may serve as a protective factor against CHD due to access to health resources, healthier lifestyle choices, and better health management.

Results further reveal that place visitation behavior of residents to selected categories of places, relevant to cardiovascular diseases (details in Section 2.3) at the census tract level, is an essential predictor of neighborhood-level CHD prevalence. 

For instance, the rate of visits to places that support physical activity engagement (such as fitness and recreational sports centers), shows a negative association with CHD prevalence. This suggests that census tract residents who visit physical activity facilities more may imply that they have higher engagement in physical activities which could lower the risk of CHD. This is consistent with existing studies [20,21]. Furthermore, good diet behavior, as measured by visitation to healthy food retail (such as Fruit and Vegetable Market), shows a reduced prevalence of CHD. This further validates findings in previous studies [57]. Conversely, a high rate of visits to less healthy food retail (such as convenience stores and limited-service restaurants), which is indicative of an unhealthy diet such as intake of fast food or junk, is associated with a high risk of CHD.

Furthermore, the results indicate that visits to drinking places (e.g., alcohol outlets) and smoking places (e.g., tobacco stores) are associated with lower CHD prevalence, an unexpected finding. This relationship may, in part, be attributed to the complex interaction between alcohol consumption and heart disease, which varies by type of beverage and drinking patterns. Some studies suggest that moderate alcohol consumption can reduce the risk of heart disease [58,59]. Additionally, this inverse association may reflect confounding by age. Statistics show that younger individuals who generally have a lower risk of CHD are more likely to engage in smoking and alcohol consumption, particularly through vaping and social drinking among high school and college-aged groups [60–62]. Considering these factors, the interaction analysis reveals that both age and income levels of residents visiting drinking places significantly influence the CHD association.

While previous studies have used place visitation data to analyze neighborhood-level health behaviors [26,28–30], our study is the first to apply it specifically to CHD prevalence at the census tract level across the US. Also, unlike traditional methods that rely on surveys and self-reported behaviors, which are often limited in scale, prone to bias, and slow to collect, this study uses passive smartphone-based mobility data, offering near real-time, large-scale insights without self-reporting biases. Furthermore, our findings reveal significant spatial heterogeneity in the association between place visitation patterns and CHD, offering more insights into how behavioral exposures vary across communities. By identifying behavior-based risk patterns at a fine geographic scale, this study supports the development of spatially targeted interventions to reduce CHD risk the leading cause of death in the US – and promote healthier communities.

### 4.1 Limitations of the study

Despite the valuable insights provided by this study, it also has significant limitations. One limitation is that the broader environmental and social determinants that influence access to health-promoting amenities are not fully accounted for. For example, neighborhoods with higher income levels may have greater access to parks, grocery stores with fresh produce, and fitness centers. There is also a well-documented pattern of unhealthy outlets, such as tobacco shops, alcohol stores, and fast-food restaurants, being disproportionately located in poorer, predominantly Black neighborhoods [63–65]. This targeted placement exacerbates the risk of CHD in these communities by making unhealthy choices more accessible and limiting exposure to healthier alternatives. For instance, studies have shown that the density of fast-food outlets is significantly higher in low-income, minority neighborhoods, leading to increased consumption of unhealthy foods and, consequently, higher CHD prevalence. These environmental and social factors have not been included in our data analysis due to the data availability issue, but they are crucial to understanding the full context in which health behaviors and CHD prevalence occur. Thus, future research should aim to incorporate measures of these determinants.

Another key limitation of this study is the inability to capture individual-level age-specific visitation behaviors, as SafeGraph data do not provide age-disaggregated place visitation details. Additionally, the CDC PLACES dataset reports CHD prevalence for individuals aged 18 and older, limiting the ability to assess CHD risk exclusively among older adults. Future research could benefit from integrating age-stratified CHD prevalence data or utilizing synthetic population modeling to refine age-specific estimates further.

Furthermore, we acknowledge that the study may have omitted certain health behaviors critical to understanding the risk of CHD, such as stress levels and access to healthcare. Although these behavioral factors significantly influence cardiovascular health, they are not directly captured in the datasets utilized for this study. However, certain socioeconomic indicators, such as low income and high unemployment may serve as proxies for stress, as financial insecurity and job instability are well-documented sources of psychological stress [66,67]. Future research may consider incorporating these indirect stress indicators or employing alternative modeling techniques that explicitly account for these variables.

Last but not least, this study used 2019 data (pre-COVID-19) in order to eliminate the impact of the pandemic. Future research could examine changes in visitation patterns across different phases of the pandemic (pre-pandemic, pandemic, and post-pandemic). Analyzing these temporal trends could offer valuable insights into how disruptions like the COVID-19 pandemic have influenced health behaviors and cardiovascular outcomes.

## 5. Conclusions

This paper investigates the potential of smartphone-based place visitation data utilization in explaining the prevalence of CHD at the neighborhood level. The association between the health behaviors derived from place visitation data and the CHD prevalence at census tract level has been explored. It was revealed that the place visitation data can provide health behavior measurements, potentially improving the estimation of neighborhood-level CHD. The derived health behavior measurements provide considerable improvement to the CHD estimation model, offering valuable insights into the association between health behaviors and CHD. Despite its limitations, this study enhances our comprehension of the potential to infer neighborhood-level health behaviors associated with CHD from anonymized smartphone-based location data, which can provide a more comprehensive explanation for the prevalence of CHD at the census tract level. Findings from this study can guide the formulation of policy and health interventions to mitigate the prevalence of CHD.

## Supporting information

S1 Data Dataset. (CSV)
